# Establishment of a genetically engineered chicken DF-1 cell line for efficient amplification of influenza viruses in the absence of trypsin

**DOI:** 10.1186/s12896-020-00663-6

**Published:** 2021-01-07

**Authors:** Kelly Chungu, Young Hyun Park, Seung Je Woo, Su Bin Lee, Deivendran Rengaraj, Hong Jo Lee, Jae Yong Han

**Affiliations:** Department of Agricultural Biotechnology and Research Institute of Agriculture and Life Sciences, College of Agriculture and Life Sciences, Seoul National University, Seoul, 08826 Korea

**Keywords:** Avian influenza virus, Chicken, DF-1 cell, Proteases, Sialic acid

## Abstract

**Background:**

The initial step of influenza infection is binding of the virus to specific sialic acid receptors expressed by host cells. This is followed by cell entry via endocytosis. Cleavage of the influenza virus hemagglutinin (HA) protein is critical for infection; this is performed by host cell proteases during viral replication. In cell culture systems, HA is cleaved by trypsin added to the culture medium. The vast majority of established cell lines are mammalian.

**Results:**

In the present study, we generated genetically engineered chicken DF-1 cell lines overexpressing transmembrane protease, serine 2 (TMPRSS2, which cleaves HA), ST3 beta-galactoside alpha-2,3-sialyltransferase 1 (ST3GAL1, which plays a role in synthesis of α-2,3 linked sialic acids to which avian-adapted viruses bind preferentially), or both. We found that overexpression of TMPRSS2 supports the virus life cycle by cleaving HA. Furthermore, we found that overexpression of ST3GAL1 increased the viral titer. Finally, we showed that overexpression of both TMPRSS2 and ST3GAL1 increased the final viral titer due to enhanced support of viral replication and prolonged viability of the cells. In addition, overexpression of these genes of interest had no effect on cell proliferation and viability.

**Conclusions:**

Taken together, the results indicate that these engineered cells could be used as a cell-based system to propagate influenza virus efficiently in the absence of trypsin. Further studies on influenza virus interactions with chicken cell host factors could be studied without the effect of trypsin on cells.

## Background

The influenza virus surface protein hemagglutinin (HA) plays two major roles during the early life cycle of the virus: it binds to cell surface receptors and facilitates fusion of viral and endosomal membranes to release viral RNA (vRNA) into the cytoplasm [1]. The HA protein is translated as an uncleaved HA0 precursor protein; it is folded as a trimer that is both glycosylated and acylated. Because uncleaved HA0 is unable to initiate membrane fusion, lack of cleavage means no infection [2, 3]. Therefore, cleavage of HA0 into HA1 and HA2 subunits is critical for membrane fusion with the endosome and subsequent release of viral segments into the cytoplasm prior to nuclear transport, transcription, and replication. Highly pathogenic avian influenza (HPAI) viruses harbor a polybasic amino acid sequence at the cleavage site, which is cleaved endogenously by ubiquitously expressed subtilisin-like proteases such as furin and proprotein convertases with polybasic specificity, resulting in fatal systemic infection [4–7]. By contrast, low pathogenic avian influenza (LPAI) viruses are cleaved by trypsin-like proteases such as miniplasmin, tryptase Clara, Mast cell tryptase, type II transmembrane serine proteases such as TMPRSS2 and TMPRSS4, and human airway trypsin-like protease (HAT) [8–10]. However, recent studies show that Madin-Darby canine kidney (MDCK) cell lines expressing proteolytic enzymes such as TMPRSS2, HAT, and Mosaic serine protease large form cleave HA in the absence of trypsin [1, 11].

Influenza viruses are propagated for vaccine production and for studies of the viral life cycle, interactions with host cellular factors, and host immune responses. Egg-based and cell-based systems are used to generate influenza vaccines. However, viruses produced in eggs often harbor undesired mutations in HA that render the vaccine less effective. In addition, some reassorted viral strains grow poorly, and highly pathogenic strains are difficult to propagate, in eggs. Other drawbacks of egg-based systems include limited flexibility for expanded vaccine manufacture and interruption of vaccine production/quality during disease outbreaks in poultry [12, 13].

Cell culture-based propagation of influenza virus is an alternative system that offers various advantages, including easy scale-up for cell engineering systems, increased vaccine purity, and utility for people with allergies to egg proteins [14]. However, propagation of LPAI viruses in cell-based culture systems requires supplementation with trypsin to cleave the HA protein and drive viral replication [15]. In some cell lines, high trypsin concentration showed also variation in the resulting yield of virus, thus requiring optimization [16]. This may be overcome by removing the reliance on exogenous trypsin. In addition, removing trypsin will reduce the costs of production. Moreover, using species specific cell lines will remove the need of viruses to adapt to host cells [16, 17].

Avian derived cell lines such as chicken DF-1 cells can be used to propagate influenza viruses because they express α-2,3-linked sialic acid receptors, which are targeted preferentially by avian-adapted viruses [18]; also, immortalized cell lines provide a suitable platform for generating stable cell lines that can be used for virus propagation. Engineering cells such that they can support faster viral replication will also be a great advantage to the vaccine industry.

Here, we developed genetically engineered chicken DF-1 cells that stably overexpress ST3 beta-galactoside alpha-2,3-sialyltransferase 1 (ST3GAL1), which catalyzes transfer of the sialic acid Neu5Ac from CMP-Neu5Ac to Galβ1,3GalNac on glycoproteins or glycolipids with an α-2,3 linkage. We also developed a cell line overexpressing type II transmembrane protease, serine 2 (TMPRSS2), which is required for cleavage of HA. Finally, we developed a cell line expressing both ST3GAL1 and TMPRSS2. The engineered cell lines allowed efficient propagation of influenza virus in the absence of exogenous trypsin. These engineered cells may provide a platform for viral amplification even in the absence of trypsin, thereby allowing development of vaccine for poultry and study of virus replication in avian cells.

## Results

### Establishment of TMPRSS2- and TMPRSS4-overexpressing cell lines and subsequent viral challenge

**Fig. 1:**
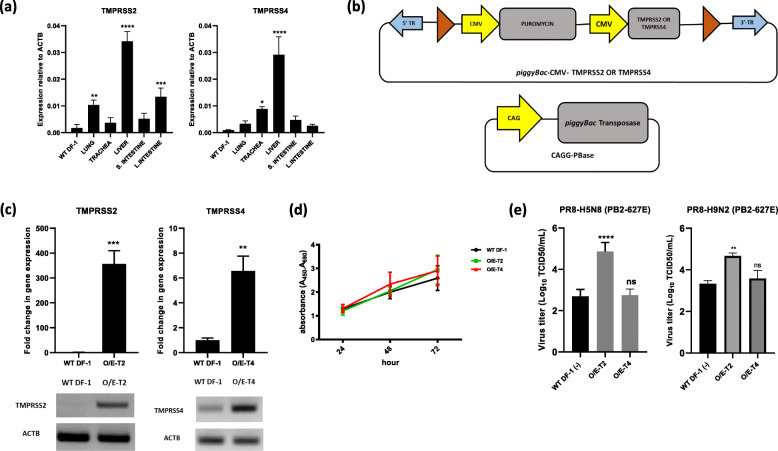
Establishment of TMPRSS2- and TMPRSS4-overexpressing cell lines and challenge with viruses. **a** Expression of TMPRSS2 and TMPRSS4 in various chicken tissues and in WT DF-1, as measured by qRT-PCR. Data are normalized to expression of chicken ACTB and are expressed as the mean ± standard deviation (*n* = 3). Significant differences (compared with WT DF-1 cells) were determined by one-way ANOVA (****P < 0.0001, ***P < 0.001, **P < 0.01, and *P < 0.05). **b** Schematic representation of the *piggyBac* transposon based expression vector harboring TMPRSS2 or TMPRSS4. The vector was used to express either TMPRSS2 or TMPRSS4 in WT DF-1 cells, termed O/E-T2 or O/E-T4. **c** (top) Expression of TMPRSS2 and TMPRSS4 in O/E-T2 and O/E-T4 and in WT DF-1 cells, as measured by qRT-PCR. Data are normalized to expression of chicken ACTB and are expressed as the mean ± standard deviation (*n* = 3). Significant differences (compared with WT DF-1 cells) were determined by Student’s t-test (***P < 0.001, **P < 0.01, and *P < 0.05). (bottom) Expression of TMPRSS2 and TMPRSS4 in O/E-T2 and O/E-T4 and in WT DF-1 cells, as measured by RT-PCR. The chicken ACTB was used as a reference gene. The full length (uncut) gel electrophoresis image is shown in Additional file 1: Fig. S1. **d** Cell proliferation at 24 h, 48 h, and 72 h after seeding. Error bars indicate the mean ± standard deviation of triplicate analyses. **e** Viral titer of PR8-H5N8 (PB2-627E) and PR8-H9N2 (PB2-627E) from O/E-T2 and O/E-T4 relative to that of WT DF-1 cells in the absence of TPCK-trypsin (WT DF-1(−)) at 24 h post-infection. Significant differences (compared with WT DF-1 cells) were determined by one-way ANOVA (****P < 0.0001, ns = no significant difference). Data are expressed as the mean ± standard deviation (*n* = 7)

The presence of trypsin-like proteases that cleave HA means that influenza viruses preferentially infect the respiratory and gastrointestinal tracts. Therefore, we compared the distribution of TMPRSS2 and TMPRSS4 in lung, trachea, liver, small intestine, and large intestine samples from WL chickens aged 18 weeks and wild-type (WT) DF-1 cells by quantitative real-time PCR (qRT-PCR). We found that compared with WT DF-1 cells, TMPRSS2 was expressed at high levels in liver, large intestine, and lung, whereas TMPRSS4 was expressed only in liver and trachea (Fig. 1a). The low expression of TMPRSS2 and TMPRSS4 by WT DF-1 cells suggests that they would not support viral replication efficiently.

Therefore, we constructed a *piggyBac* transposon vector that contained the protein coding region for either chicken TMPRSS2 or TMPRSS4 (Fig. 1b). This was used to drive overexpression in DF-1 cells. We then measured the effects on cell viability and proliferation of influenza virus. Firstly, we analyzed expression of mRNA in genetically engineered cells. The qRT-PCR results showed that overexpression of TMPRSS2 mRNA in TMPRSS2 overexpressing (O/E-T2) cells was 350-fold higher than that in WT DF-1 cells, whereas expression of TMPRSS4 mRNA in O/E-T4 cells was about 6-fold higher than that in WT DF-1. Reverse transcription PCR (RT-PCR) was conducted to further verify the expression of TMPRSS2 and TMPRSS4 (Fig. 1c). To assess whether overexpression of TMPRSS2 and TMPRSS4 had an antagonistic effect on cell proliferation and viability, we conducted a cell proliferation assay. The results showed that proliferation of genetically engineered cells was comparable with that of WT DF-1 cells (Fig. 1d).

Subsequently, we asked whether the proteolytic activity of TMPRSS2 and TMPRSS4 supports viral infectivity and the viral life cycle. Engineered cells were infected with PR8-H5N8 (PB2-627E) and PR8-H9N2 (PB2-627E) [multiplicity of infection (MOI) = 0.1] in the absence of trypsin and the median tissue culture infectious dose (TCID_50_) was calculated to determine the viral titer. Notably, the viral titer in O/E-T2 cells was 35-fold higher when infected with PR8-H5N8 and 23-fold higher when infected with PR8-H9N2 than that in WT DF-1 cells, indicating proteolytic activation of HA by TMPRSS2 and subsequent support of viral replication. Thus, the cell line is suitable for amplification of influenza virus. However, we found no significant difference in the viral titer between O/E-T4 cells overexpressing TMPRSS4 protease and WT-DF1 cells (Fig. 1e) for both strain of viruses.

### Establishment of ST3GAL1-overexpressing cells and determination of viral titer

**Fig. 2:**
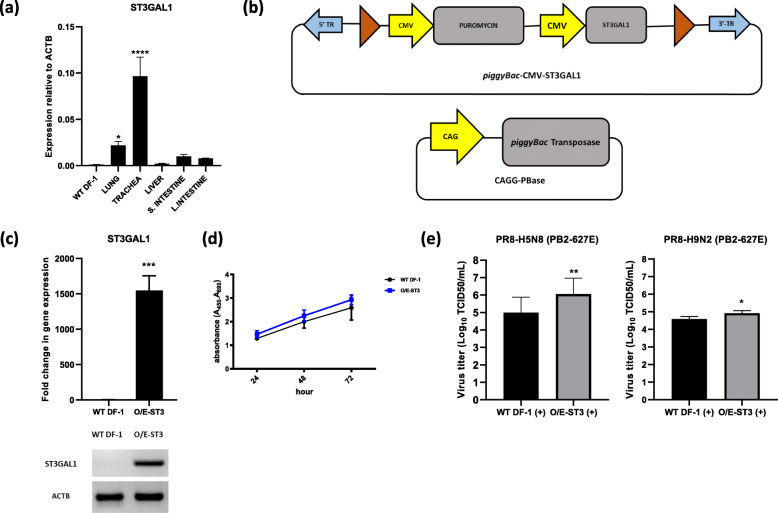
Establishment of ST3GAL1-overexpressing cell lines and challenge with viruses. **a** Comparison of ST3GAL1 expression in chicken tissues and WT DF-1 cells by qRT-PCR. Data were normalized to expression of chicken ACTB and are expressed as the mean ± standard deviation (*n* = 3). Significant differences (compared with WT DF-1 cells) were determined by one-way ANOVA (****P < 0.0001, and *P < 0.05). **b** Schematic representation of the *piggyBac* transposon based expression vector harboring ST3GAL1. The vector was used to express ST3GAL1 in WT DF-1 cells, termed O/E-ST3. **c** (top) Expression of ST3GAL1 in O/E-ST3 and WT DF-1 cells, as detected by qRT-PCR. Data were normalized to expression of chicken ACTB and are expressed as the mean ± standard deviation (*n =* 3). Significant differences (compared with WT DF-1 cells) were determined by Student’s t-test (***P < 0.001, **P < 0.01, and *P < 0.05) (bottom) Expression of ST3GAL1 in O/E-ST3 and in WT DF-1 cells, as measured by RT-PCR. The chicken ACTB was used as a reference gene. The full length (uncut) gel electrophoresis image is shown in Additional file 2: Fig. S2. **d** Cell proliferation at 24 h, 48 h, and 72 h. Error bars indicate the mean ± standard deviation of triplicate analyses. **e** Relative titer of PR8-H5N8 (PB2-627E) or PR8-H9N2 (PB2-627E) after treatment with trypsin (+) in O/E-ST3 cells (O/E-ST3(+)) compared with that in WT DF-1 (WT DF-1(+)) cells treated with trypsin at 24 h post-infection. Significant differences were determined by Student’s t-test (**P < 0.01 and *P < 0.05). Error bars indicate the mean ± standard deviation of triplicate analyses

Sialic acid residues on cell surface receptors are important for binding and endocytosis of influenza virus. Therefore, we examined expression of ST3GAL1 in lung, trachea, liver, small intestine, and large intestine samples from WL chickens and compared it with that by WT DF-1 using qRT-PCR. The results revealed that expression of ST3GAL1 in trachea and lung was significantly higher than that by WT DF-1 cells (Fig. 2a).

Since WT DF-1 cells expressed lower levels of ST3GAL1, we constructed a *piggyBac* transposon vector containing the protein coding sequence of chicken ST3GAL1 (Fig. 2b) and transfected it into WT DF-1 to engineer cells that express high levels of ST3GAL1. Subsequently, we analyzed expression of ST3GAL1 in ST3GAL1 overexpressing (O/E-ST3) cells by qRT-PCR. The results showed a 1500-fold increase in expression compared with that in WT DF-1 cells. RT-PCR was conducted to further verify the expression ST3GAL1 (Fig. 2c). To access whether cells overexpressing ST3GAL1 had an antagonistic effect on cell proliferation and viability, we performed a cell proliferation assay. The results revealed that proliferation of genetically engineered cells was comparable with that of WT DF-1 cells (Fig. 2d).

Finally, to examine whether increased expression of ST3GAL1 correlates positively with viral titer, O/E-ST3 cells were infected with PR8-H5N8 (PB2E-627E) or with PR8-H9N2 (PB-627E) at MOI of 0.1 in the presence of trypsin. The TCID_50_ was calculated to determine the viral titer. The results showed the viral titer in O/E-ST3 cells was significantly higher with both strain of viruses than that in WT DF-1 cells (Fig. 2e).

### Establishment of cell lines expressing both ST3GAL1 and TMPRSS2 and their effect on virus titers

**Fig. 3:**
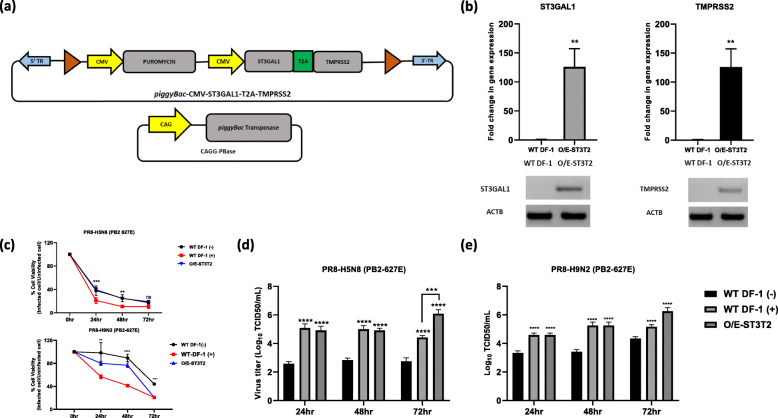
Combined overexpression of ST3GAL1 and TMPRSS2 and the resulting viral titer in cells. **a** Schematic representation of the *piggyBac* transposon based expression vector harboring ST3GAL1-*T2A*-TMPRSS2. The vector was used to express both ST3GAL1 and TMPRSS2 in WT DF-1 cells, termed O/E-ST3T2. **b** (top) Expression of ST3GAL1 and TMPRSS2 in O/E-ST3T2 and WT DF-1 cells was analyzed by qRT-PCR. Data were normalized to expression of chicken ACTB and expressed as the mean ± standard deviation (*n =* 3). Significant differences (compared with WT DF-1 cells) were determined using Student’s t-test (**P < 0.01 and *P < 0.05). (bottom). Expression of ST3GAL1 and TMPRSS2 in O/E-ST3T2 and in WT DF-1 cells, as measured by RT-PCR. The chicken ACTB was used as a reference gene. The full length (uncut) gel electrophoresis image is shown in Additional file 3: Fig. S3. **c** Titer of PR8-H5N8 (PB2-627E) or PR8-H9N2 (PB2-627E) in O/E-ST3T2 cells and WT DF-1 cells in the absence (WT DF-1(−)) and presence (WT DF-1(+)) of trypsin. **d** Cell proliferation at 24 h, 48 h, and 72 h post-infection. Error bars indicate the mean ± standard deviation of triplicate analyses. **e** Viral titer at 24 h, 48 h and 72 h. Significant differences (compared with WT DF-1 cells) were determined by two-way ANOVA. A *P* value < 0.05 was considered significant (****P < 0.0001 and ***P < 0.001). Error bars indicate the mean ± standard deviation of triplicate analyses

To further investigate whether overexpressing both ST3GAL1 and TMPRSS2 (O/E-ST3T2) generates higher viral titers, we constructed a new *piggyBac* transposon vector containing the protein coding sequences of ST3GAL1 and TMPRSS2 linked by the self-cleaving peptide T2A (Fig. 3a). Cells were transfected with the overexpression vector and analyzed by qRT-PCR. The results showed a 120-fold increase in expression of both ST3GAL1 and TMPRSS2. RT-PCR was conducted to verify the expression of both ST3GAL1 and TMPRSS2 in O/E-ST3T2 (Fig. 3b).

To understand the effect of trypsin on cell viability after infection by influenza virus, we performed a cell proliferation assay. The results revealed a significant difference in viability between WT DF-1(+) and O/E-ST3T2 cells at 24 h and 48 h post-infection, but not at 72 h post-infection (Fig. 3c) with PR8-H5N8 (PB2-627E). However, with PR8-H9N2 cell viability was very gradual except in WT DF-1 treated with trypsin.

Finally, we examined viral titers at different time points post-infection. Intriguingly, the viral titer in O/E-ST3T2 cells was significantly higher than that in WT DF-1(+) transfected with empty plasmid control at 72 h post-infection infected with either PR8-H5N8 (PB2-627E) (Fig. 3d) or PR8-H9N2 (PB2-627E) (Fig. 3e). Taken together, the results indicate that engineered O/E-ST3T2 cells were able to produce high viral titers in the absence of trypsin due to prolonged viability.

## Discussion

Vaccine production in egg-based systems has many disadvantages [12, 13]; therefore, cell-based systems are a suitable alternative. Several cell lines have been established [19] to meet different parameters for virus amplification. However, cell-based systems require addition of exogenous trypsin to support viral replication. Furthermore, developing cell lines from different species is beneficial as it will obviate the need for virus adaptation.

Here, we established chicken DF-1 cells overexpressing chicken protease TMPRSS2; this means that they do not require addition of exogenous trypsin for virus amplification. Consistent with other reports [1, 8, 20], we found that cells expressing TMPRSS2 supported influenza replication without the need for trypsin. Studies show that HA is cleaved by TMPRSS2 at the plasma membrane during post-translational modification [21]. However, with respect to expression of TMPRSS4, we speculated that cells may not support viral replication in the absence of trypsin. Interestingly, overexpression of proteases was not toxic to cells, which grew as well as WT DF-1 cells, even after several serial passages.

Increased expression of sialic acid receptors on the cell surface increases uptake of influenza viruses by cells. Therefore, we engineered cells to overexpress ST3GAL1. These cells generated high virus titers at 24 h post-infection with the addition of trypsin, suggesting that high expression of α-2,3 linked sialic acid residues increased viral uptake. These results are consistent with those of other studies suggesting that abundant expression of sialic acid increases infection of host cells by influenza viruses [22, 23].

Finally, we showed that cells overexpressing both ST3GAL1 and TMPRSS2 generated greater viral titers than WT DF-1 cells treated with trypsin. Intriguingly, at 24 h and 48 h post-infection, virus titers were comparable with those in WT DF-1 cells treated with trypsin. However, the viability of overexpressing cells was superior at later time points, suggesting that cell viability had an effect on the final viral titers. As expected, viral titers were highest in O/E-ST3T2 cells at 72 h post-infection; this was most likely due to prolonged viability.

## Conclusions

We showed that genetically engineered chicken DF-1 cells overexpressing both TMPRSS2 and ST3GAL1 support infection and replication of influenza virus in the absence of trypsin. These cell lines could be useful for studying influenza virus replication and host responses in the absence of trypsin, which can degrade interferons secreted by cultured cells [24]. Furthermore, this system can be adapted to amplify influenza for vaccine production because avian-adapted influenza viruses do not require cell adaptation. Collectively, these cell lines can be added to the growing pool of cell-based systems useful for influenza virus amplification.

## Methods

### Experimental animals and tissue collection

The management and experimental use of White Leghorn (WL) chickens and cell lines were approved by the Institutional Animal Care and Use Committee, Seoul National University (SNU-190401-1). The experimental animals were cared for at the University Animal Farm, Seoul National University, in accordance with standard management programs. The chickens used in this study were euthanized by placing in CO_2_ chamber, then carbon dioxide was filled until the chickens were unconscious and showed no signs of life. After five minutes, the tissue samples from adult WL chicken aged 18 weeks were collected.

### Viruses and biosafety

The PR8-H5N8 (PB2-627E) and PR8-H9N2 (PB2-627E) low pathogenic viruses were generated from eight bidirectional pHW2000 plasmids (a kind gift from Prof. Song Chang Seon of Konkuk University, South Korea) using a reverse genetics system, as previously reported [25]. These viruses are an avian adapted strains (PB2-627E), and therefore, they are suitable to use as the proof of concept. Viruses were rescued by co-transfection of the eight bidirectional plasmids into a co-culture of MDCK cells (MDCK (NBL-2), CCL-34, ATCC) and human 293 T embryonic kidney cells (293 T, CRL-11268, ATCC). Generated viruses were grown in MDCK infection medium comprising Dulbecco’s Modified Eagle’s Medium (DMEM) (Hyclone, Logan, UT, USA) supplemented with 0.3% bovine serum albumin (BSA), 1× antibiotic antimycotic (ABAM), and 1 μg/ml L-(tosylamido-2-phenyl) ethyl chloromethyl ketone (TPCK)-treated trypsin (Sigma-Aldrich, MO, USA), and then incubated at 37 °C for 48 h. Virus stocks were further propagated in 10-day-old embryonated chicken eggs. Aliquots of infectious virus were stored at − 80 °C until required. All work with low pathogenicity viruses was conducted in a biosafety level 2 facility approved by the Institutional Biosafety Committee of Seoul National University.

### Construction of overexpression plasmids

The *piggyBac* plasmid (Addgene plasmid no. 92078, Addgene, MA, USA) containing enhanced green fluorescent protein (eGFP) was digested with AgeI and BsrGI enzymes to create a linearized vector. A synthetic protein coding region of chicken TMPRSS2 (NCBI Gene ID 418528), chicken TMPRSS4 (NCBI Gene ID 770454), chicken ST3GAL1 (NCBI Gene ID 396140), or chicken ST3GAL1-T2A-TMPRSS2 (Bionics, Korea) was cloned into the linearized vector using Takara In-Fusion Ligation mix (Takara, Kasatsu, Japan), according to the manufacturer’s protocol. The resulting plasmid was amplified and purified using a Plasmid Maxi kit (Qiagen, Hilden, Germany). The correct insert was confirmed by sequencing.

### Cell culture and establishment of overexpressing DF-1 cell lines

Chicken DF-1 fibroblast cells (UMNSAH/DF-1, CRL-12203, ATCC) were maintained in high glucose DMEM supplemented with 10% fetal bovine serum (Hyclone) and 1× ABAM. Cells were maintained at 37 °C at 5% CO_2_ under 60–70% relative humidity. To establish cell lines that stably overexpress the genes of interest, 1.2 μg of overexpression vector and 0.8 μg of *piggyBac* transposon (pCyL50) were transfected into DF-1 cells using Lipofectamine 2000 (Thermo Fisher Scientific, Waltham, MA, USA). After 6 h, transfection mixtures were replaced with DF-1 culture medium supplemented with puromycin (Thermo Fisher Scientific). Cells were maintained in culture medium supplemented with puromycin for 1 week to recover overexpressing cells. The cell lines were maintained for more than 15 passages for subsequent analysis.

### RNA isolation, qRT-PCR and RT-PCR analysis

Total RNA from cells or tissues was extracted using Tri-reagent (Molecular Research Center Inc., Cincinnati, Ohio, USA). RNA quantity was determined by spectrophotometry at 260 nm, and 0.5–1 μg of each sample was reverse-transcribed using the Superscript IV First-Strand Synthesis System (Thermo Fisher Scientific). The complementary DNA (cDNA) was diluted five-fold and the concentrations standardized for amplification by PCR. qRT-PCR was conducted to examine changes in expression of candidate genes in test samples and in overexpressing DF-1 cells. The PCR reaction mixture contained 2 μl of PCR buffer, 1 μl of 20× EvaGreen qPCR dye (Biotium, Hayward, CA, USA), 0.5 μl of 10 mM dNTP mixture, 10 pmole each of target gene-specific forward and reverse primers (Table 1), 1 μl of cDNA, and 1 U of *Taq* DNA polymerase (final volume, 20 μl). qRT-PCR was conducted in a StepOnePlus real-time PCR system (Applied Biosystems, CA, USA). Each test sample was assayed in triplicate. Relative quantification of target gene expression in the cells was performed using the following formulae: 2^–ΔCt^, where ΔCt = (Ct of the target *gene*–Ct of *ACTB*), or 2^–ΔΔCt^, where ΔΔCt = (Ct of the target *gene*–Ct of *ACTB*) group–(Ct of the target *gene*–Ct of *ACTB*) control. RT-PCR was conducted to examine changes in expression of candidate genes in test samples and in overexpressing DF-1 cells. The PCR reaction mixture contained 2 μl of PCR buffer, 0.5 μl of 10 mM dNTP mixture, 10 pmole each of target gene-specific forward and reverse primers (Table 1), 1 μl of cDNA, and 1 U of *Taq* DNA polymerase (final volume, 20 μl). RT-PCR was performed using T100 Thermal Cycler (Bio-Rad, Hercules, CA, USA), and the products were separated by gel electrophoresis and visualized using the Gel Doc XR+ Imaging System (Bio-Rad). The chicken ACTB was used as a reference gene.

**Table 1:** List of primers used for qRT-PCR and RT-PCR

ID	Sequence (5′ → 3′)
ACTB - F	AGGAGATCACAGCCCTGGCA
ACTB - R	CAATGGAGGGTCCGGATTCA
ST3GAL1 - F	CACCCACCATTGGCTACGAA
ST3GAL1 - R	AGGCCTGTGGAAGGGTATCT
TMPRSS2 - F	TTCTGCCAGGCCACAAGTAG
TMPRSS2 - R	GGAGAAATGCACACTCCCGA
TMPRSS4 - F	TCCCCTCTGGATCCTCACTG
TMPRSS4 - R	TCCAGCTCCTCGTCGAAGTA

### Viral infection of cells

The O/E-ST3, O/E-T2, O/E-ST3T2, or WT DF-1 cells were seeded on 12-well plates and grown to confluence. The culture medium was removed and the cells were washed twice with PBS prior to incubation at 37 °C for 20 min in DMEM containing 1% penicillin/streptomycin. Next, DMEM containing 1% penicillin/streptomycin containing PR8-H5N8 (PB2-627E) or with PR8-H9N2 (PB2-627E) virus at a multiplicity of infection (MOI) of 0.1 or 0.01 was added for 50 min. Finally, cells were washed with PBS, and incubated with DMEM containing 1% penicillin/streptomycin for 24 h, 48 h, and 72 h until harvesting the medium for viral titration as stated below.

### WST-1 assay of overexpressing cells and virus-infected cells

The Premix WST-1 Cell Proliferation Assay System (Takara Bio, Kusatsu, Japan) was used to measure cell proliferation. Briefly, cells (0.15 × 10^4^ cells per well) were seeded in a 96-well plate in which each well contained 0.1 ml of culture medium. At 2 h before each time point (24 h, 48 h and 72 h) post-seeding, 10 μl of WST-1 Premix solution was added to the cells and incubated at 37 °C. Next, optical density was measured at an absorbance of 450 and 690 nm (A450–A690). Data were analyzed to determine proliferation and viability. Similarly, infected O/E-ST3, O/E-T2, and O/E-ST3T2 cells (1 × 10^4^ cells per well) were seeded in a 96-well plate in which each well contained 0.1 ml of culture medium. One day later, confluent DF-1 cells were infected with PR8-H5N8 (PB2-627E) or with PR8-H9N2 (PB2-627E) at a MOI of 0.1. Percentage survival was calculated as the ratio of the optical absorbance at 450 and 690 nm (A450–A690) of infected DF-1 cells and non-infected control DF-1 cells. All experiments were performed in triplicate, with three independent samples.

### Viral titration

The virus titer in infected O/E-ST3, O/E-T2, O/E-ST3T2 DF-1 cells, or WT DF-1 cells was determined by calculating the median tissue culture infectious dose (TCID_50_). MDCK cells were cultured in Eagle’s Minimum Essential Medium (EMEM) (ATCC, Manassas, VA, USA) supplemented with 10% fetal bovine serum (Hyclone) and 1x ABAM. For viral titration, MDCK cells (2.5 × 10^4^ cells per well) were seeded in 96-well plates until confluence in culture media and subsequently confluent layer of MDCK cells were wash with PBS and treated with DMEM supplemented with 1% penicillin/streptomycin and infected with PR8-H5N8 (PB2-627E) or with PR8 H9N2 (PB2-627E) from the O/E-ST3, O/E-T2, O/E-ST3T2 DF-1 cells, or WT DF-1 cells for 50 min at 37 °C. Then, the MDCK cells was washed with PBS, and finally incubated with DMEM supplemented with 0.3% BSA, 1% penicillin/streptomycin, and 1 μg/ml TPCK-trypsin at 37 °C. After 72–96 h, the plate was stained with crystal violet (Sigma-Aldrich) to observe cytopathic effects. The TCID_50_ values per milliliter were calculated using the Spearman-Karber formula [26].

### Statistical analysis

Statistical analysis was performed using GraphPad Prism software (GraphPad Software 8, San Diego, CA). Significant differences between two groups were determined using Student’s t-test. Significant differences between groups were determined using ANOVA with Bonferroni’s multiple comparison. A *P* value < 0.05 was considered significant (****P < 0.0001, ***P < 0.001, **P < 0.01, and *P < 0.05).

## Supplementary Information

**Additional file 1: Figure S1.** The full length (uncut) gel electrophoresis image of Fig. 1c.

**Additional file 2: Figure S2.** The full length (uncut) gel electrophoresis image of Fig. 2c.

**Additional file 3: Figure S3.** The full length (uncut) gel electrophoresis image of Fig. 3b.

## Data Availability

The datasets used and/or analyzed during the current study are available from the corresponding author on reasonable request.

## Abbreviations

HA: Hemagglutinin
HAT: Human airway trypsin-like protease
HPAIV: High pathogenic avian influenza virus
LPAIV: Low pathogenic avian influenza virus
MDCK: Madin-darby canine kidney
ST3GAL1: ST3 beta-galactoside alpha-2,3-sialyltransferase 1
TMPRSS2: Transmembrane serine protease 2
TMPRSS4: Transmembrane serine protease 4
O/E-ST3: Overexpression of ST3 beta-galactoside alpha-2,3-sialyltransferase 1
O/E-ST3T2: Overexpression of both ST3 beta-galactoside alpha-2,3-sialyltransferase 1 and transmembrane serine protease 2
O/E-T2: Overexpression of transmembrane serine protease 2
O/E-T4: Overexpression of transmembrane serine protease 4
vRNA: Viral ribonucleic acid
