# Supplementary figures and images for: Establishment of a genetically engineered chicken DF-1 cell line for efficient amplification of influenza viruses in the absence of trypsin
Source: BMC Biotechnol. 2021 Jan 7;21:2. doi: 10.1186/s12896-020-00663-6 (PMC7792337; doi:10.1186/s12896-020-00663-6)

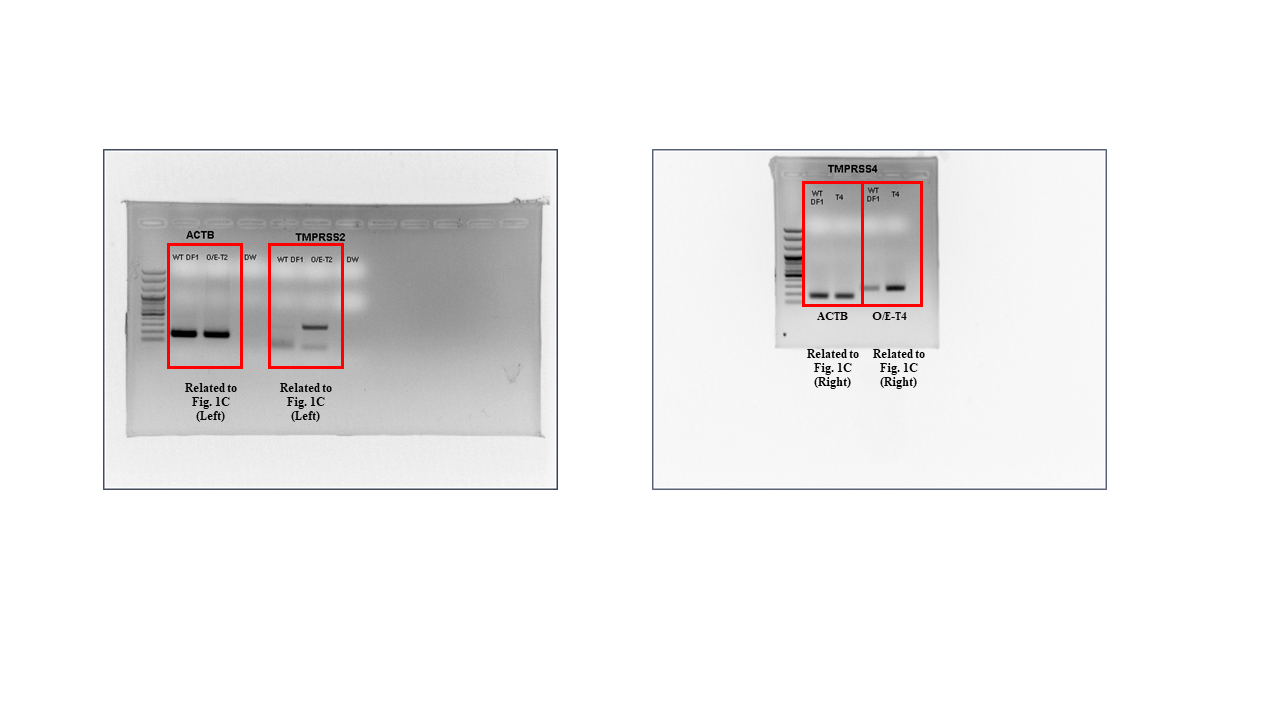

Supplement: Supplementary file 1 — Additional file 1: Figure S1. The full length (uncut) gel electrophoresis image of Fig. 1c. [file 12896_2020_663_MOESM1_ESM.tif]

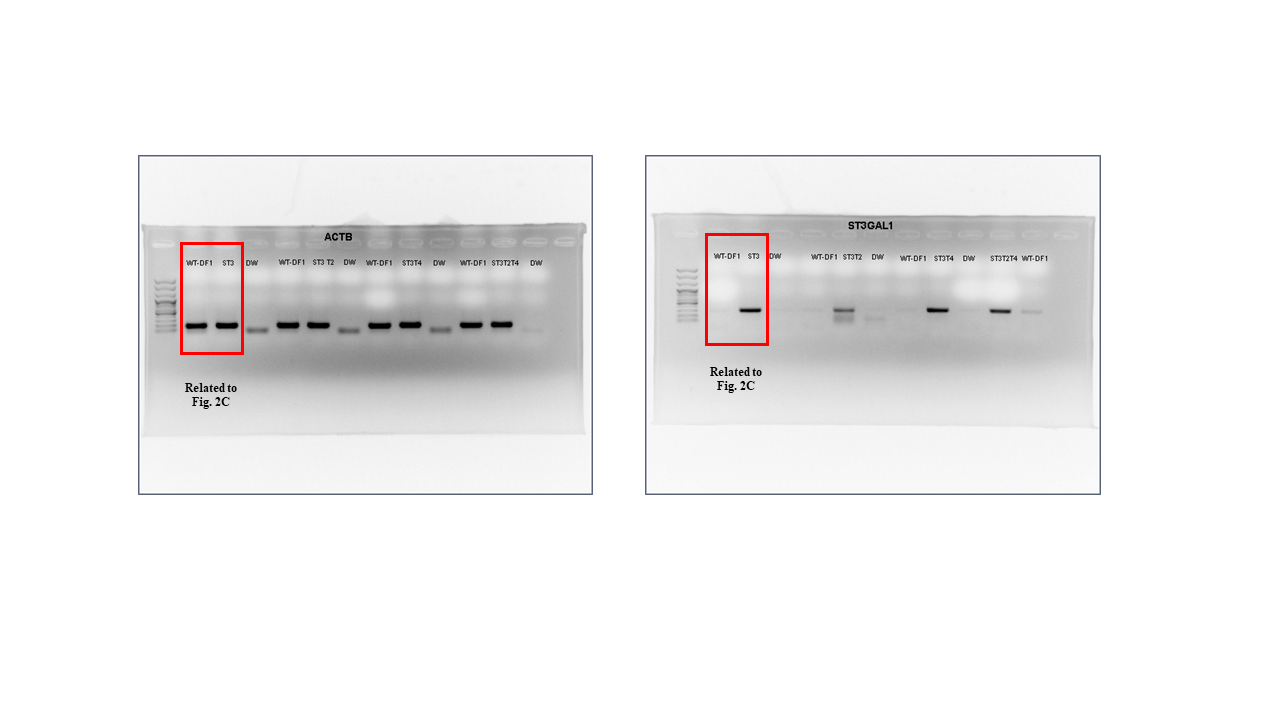

Supplement: Supplementary file 2 — Additional file 2: Figure S2. The full length (uncut) gel electrophoresis image of Fig. 2c. [file 12896_2020_663_MOESM2_ESM.tif]

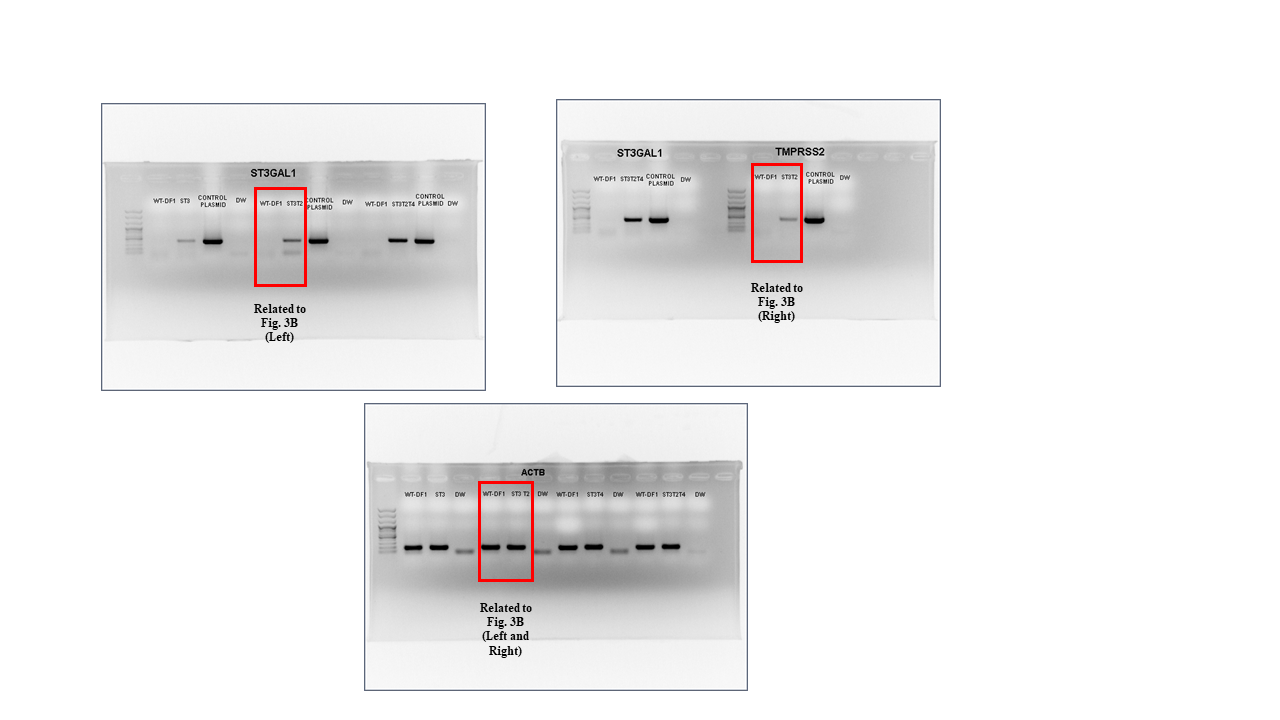

Supplement: Supplementary file 3 — Additional file 3: Figure S3. The full length (uncut) gel electrophoresis image of Fig. 3b. [file 12896_2020_663_MOESM3_ESM.tif]
